# Aberrant *Zip14* expression in muscle is associated with cachexia in a *Bard1*‐deficient mouse model of breast cancer metastasis

**DOI:** 10.1002/cam4.3242

**Published:** 2020-07-30

**Authors:** Ahmad Rushdi Shakri, Timothy James Zhong, Wanchao Ma, Courtney Coker, Rohaan Hegde, Hanna Scholze, Vanessa Chin, Matthias Szabolcs, Hanina Hibshoosh, Kurenai Tanji, Richard Baer, Anup Kumar Biswas, Swarnali Acharyya

**Affiliations:** ^1^ Institute for Cancer Genetics Columbia University New York NY USA; ^2^ Graduate School of Arts and Sciences Department of Pathobiology and Mechanisms of Disease Columbia University Irving Medical Center New York NY USA; ^3^ Department of Biological Sciences Columbia University New York NY USA; ^4^ Barnard College Columbia University New York NY USA; ^5^ Weill Cornell Medical College New York NY USA; ^6^ Department of Pathology and Cell Biology Columbia University New York NY USA; ^7^ Division of Neuropathology Department of Pathology and Cell Biology Columbia University Medical Center and New York Presbyterian Hospital New York NY USA; ^8^ Herbert Irving Comprehensive Cancer Center Columbia University New York NY USA

**Keywords:** BRCA mutations, breast cancer, cachexia, metastasis, mouse models

## Abstract

Nearly 80% of advanced cancer patients are afflicted with cachexia, a debilitating syndrome characterized by extensive loss of muscle mass and function. Cachectic cancer patients have a reduced tolerance to antineoplastic therapies and often succumb to premature death from the wasting of respiratory and cardiac muscles. Since there are no available treatments for cachexia, it is imperative to understand the mechanisms that drive cachexia in order to devise effective strategies to treat it. Although 25% of metastatic breast cancer patients develop symptoms of muscle wasting, mechanistic studies of breast cancer cachexia have been hampered by a lack of experimental models. Using tumor cells deficient for BARD1, a subunit of the BRCA1/BARD1 tumor suppressor complex, we have developed a new orthotopic model of triple‐negative breast cancer that spontaneously metastasizes to the lung and leads to systemic muscle deterioration. We show that expression of the metal‐ion transporter, *Zip14*, is markedly upregulated in cachectic muscles from these mice and is associated with elevated intramuscular zinc and iron levels. Aberrant *Zip14* expression and altered metal‐ion homeostasis could therefore represent an underlying mechanism of cachexia development in human patients with triple‐negative breast cancer. Our study provides a unique model for studying breast cancer cachexia and identifies a potential therapeutic target for its treatment.

## Graphical abstract

In this study, we have developed a new orthotopic model of triple‐negative breast cancer that spontaneously metastasizes to the lung and leads to systemic muscle deterioration. We show that aberrant Zip14 expression and altered metal‐ion homeostasis represent an underlying mechanism of cachexia development in this triple‐negative breast cancer model.

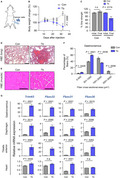

## 1 INTRODUCTION

Cachexia is a debilitating syndrome characterized by a progressive loss of muscle mass and function.^1^ Cachectic cancer patients also exhibit systemic metabolic dysfunction, adipose tissue wasting, and chronic inflammation.^2^, ^3^, ^4^ Moreover, the reduced synthesis and increased degradation of muscle proteins coupled with a systemic increase in energy expenditure lead to the wasting of both cardiac and skeletal muscles in cancer patients.^5^, ^6^ As such, these patients suffer from a disruption of vital physiological functions that are controlled by muscles, such as breathing, swallowing, pumping blood, and locomotion, and therefore exhibit a higher risk of succumbing to respiratory and cardiac failure from skeletal and cardiac muscle wasting.^7^ Cachectic cancer patients also display a decreased tolerance to antineoplastic therapy, often necessitating either dose reduction or the discontinuation of treatment, that worsens their prognosis and further diminishes their likelihood of survival.^5^ Although cachexia affects almost 80% of advanced cancer patients, no approved anticachexia treatments have been developed.^3^, ^6^, ^8^ As a result, the presence of cachexia remains a harbinger of poor quality of life and reduced survival that is independent of cancer type.^2^, ^3^, ^9^

Since breast cancer is not typically associated with the loss of body weight, cachexia has historically been underdiagnosed in breast cancer patients.^2^, ^3^ Instead, obesity or weight gain is often observed and correlates with a higher recurrence of tumors and reduced survival.^10^, ^11^, ^12^ However, recent advances in computed tomography (CT)‐based body composition analysis^13^ have revealed that skeletal muscle wasting is often masked in breast cancer patients by excess adipose tissue (due to obesity or anticancer treatments).^14^, ^15^, ^16^, ^17^, ^18^, ^19^ Imaging studies by the Prado group confirmed that age‐related loss of muscle mass and decline in physical strength and functional ability (known as sarcopenia) occur in 25% of metastatic breast cancer patients and are the significant predictors of toxicity to antineoplastic agents and time to tumor progression.^14^, ^16^, ^19^ Additional studies observed muscle weakness and body pain in 39% of breast cancer patients, leading to functional limitations and increased morbidity.^20^, ^21^ Therefore, muscle health is clearly a critical determinant of survival, quality of life, and tolerance to antineoplastic therapy in breast cancer patients.

To understand the mechanisms of muscle wasting in breast cancer, it is important to generate models that exhibit the full spectrum of muscle wasting states that are associated with the human disease. Patient‐derived xenograft models from early stage breast cancers^22^ or advanced‐stage breast cancers with bone metastasis^23^ have been generated that develop skeletal muscle dysfunction or fatigue without muscle mass loss, but the availability of breast cancer mouse models that recapitulate both muscle mass loss and dysfunction is limited.^24^, ^25^ This limitation has created a bottleneck for necessary mechanistic studies in the field of breast cancer cachexia. In this study, we report a new metastatic model of murine triple‐negative breast cancer that develops cachexia with loss of both muscle mass and function.

Women who carry germline mutations of the *BRCA1* tumor suppressor gene are at high risk for basal‐like, triple‐negative breast cancer.^26^ The wild‐type protein product of *BRCA1* interacts with the related BARD1 protein to form the BRCA1/BARD1 heterodimer,^27^ a central mediator of the DNA damage response pathway.^28^ Germline mutations of *BARD1* have also been implicated as pathogenic lesions in some families with hereditary breast cancer,^29^ and conditional inactivation of either *Brca1* or *Bard1* in mice generates mammary carcinomas that closely resemble the basal‐like, triple‐negative breast cancers that arise in human BRCA1 mutation carriers.^30^, ^31^ Here, we generated a cachexia‐associated breast cancer metastasis model using murine breast cancer cells from the *Bard1*‐deficient, genetically engineered mouse model.^30^ We show that upon orthotopic injection into the mammary fat pad of mice, *Bard1‐*deficient cells spontaneously metastasize to the lung and induce cachexia in multiple muscles. Studies have shown that the metal‐ion transporter, solute carrier family 39 member 14 (SLC39A14, also known as ZIP14), is a key mediator of cachexia in metastatic cancers.^25^ ZIP14 upregulation promotes cachexia through increased zinc influx in muscle cells, leading to myosin heavy chain loss in mature muscle cells and the inhibition of muscle‐progenitor‐cell differentiation. Here, we report that *Zip14* upregulation and altered metal‐ion homeostasis are likely to underlie the development of cachexia in the *Bard1*‐deficient, orthotopic mouse model of breast cancer metastasis, a finding that could have clinical implications for triple‐negative breast cancers with BRCA1/BARD1 dysfunction.

## 2 METHODS

### 2.1 Animal studies

All animal protocols and treatment of mice were approved by the Columbia University Institutional Animal Care and Use Committee (IACUC), and all animal experiments were conducted according to the ethical regulations described in the institutional guidelines of Columbia University Irving Medical Center (CUIMC), in compliance with the US National Research Council's Guide for the Care and Use of Laboratory Animals, the US Public Health Service Policy on Humane Care and Use of Laboratory Animals, and the Guide for the Care and Use of Laboratory Animals. Mice were maintained in the CUIMC barrier facility under conventional conditions with constant temperature and humidity and fed a standard diet (Labdiet 5053). Female, 8‐ to 9‐week‐old B6129SF1/J mice were obtained from the Jackson Laboratory (Bar Harbor, Maine). Mice were injected with 5 × 10^5^ *Bard1*‐deficient murine breast cancer cells orthotopically into the left fourth mammary fat pad. Mouse whole body weight and tumor volume were measured weekly. The length and width of tumors were measured using digital calipers, and tumor volume was determined by the formula: Volume (mm^3^) = (*W* ^2^) × *L*/2 (*W* = width; *L* = length). Mouse body condition as an indicator of cachexia was determined by observation of mice, as previously described.^32^

### 2.2 Body‐weight and grip‐strength measurements

Body weights of control and tumor‐bearing mice were measured weekly using a digital weighing balance. Body weights were normalized to the initial body weight measurement on the day of injection (day 0, 100%) and converted to percent body weight change for subsequent measurements. Tumors were only excised at end point for collection and whole body weights included weight of the tumor at all time points. Hind limb grip strength from control and tumor‐bearing mice was measured using a digital grip strength meter (Columbus Instruments). Five measurements of hind limb grip strength were performed for each mouse per time point, and mean values were determined from replicate readings. Percent grip strength was determined by normalizing grip strength values to the mean of the initial values for the respective groups taken on the day of injection (day 0, 100%).

### 2.3 Cell culture

*Bard1‐*deficient murine mammary cancer cells were isolated and derived from a primary mammary tumor of a *Bard1* mammary‐specific conditional mutant (Bard1^flex1/flex1^/ Wap^cre/+^) mouse with a B6129SF1/J background by the Baer laboratory.^30^ *Bard1‐*deficient murine mammary cancer cells were cultured in DMEM supplemented with 10% fetal bovine serum and grown at 37°C in a humidified incubator (5% CO_2_). All media was supplemented with 100 IU/mL penicillin and 100 μg/mL streptomycin (Life Technologies).

### 2.4 Tissue collection

Mice were euthanized according to the methods described in approved animal study protocols. Gastrocnemius, tibialis anterior, diaphragm, and heart muscles were collected in 4% paraformaldehyde for histology or snap frozen in liquid nitrogen for molecular analysis. Snap frozen samples were stored at −80°C for subsequent molecular analysis. Liver and lungs were also collected in 4% paraformaldehyde. All samples collected in paraformaldehyde were fixed for 24 hours, washed in PBS for an hour, and stored in 70% ethanol until subsequent histological processing and analysis.

### 2.5 Histological analysis of lung tissues 

Lungs were fixed in paraformaldehyde, embedded in paraffin blocks, and 5 µm sections were stained with hematoxylin and eosin (H&E). Stained sections were visualized under 20× magnification for the presence of lung metastases under a DM5500B microscope (Leica). Representative images were taken at 20× magnification.

### 2.6 Muscle cross‐sectional area quantification

Gastrocnemius muscles from control and tumor‐bearing mice were embedded in paraffin blocks, and 5 µm sections were stained with H&E. Images of muscle cross sections were taken at 20× magnification and used for muscle fiber cross‐sectional area (CSA) quantification using ImageJ software (Version 1.52). CSAs of individual myofibers (average of 250 fibers per mouse sample) were measured by marking the boundary of each fiber using a drawing tool in ImageJ. Fibers were stratified into various area ranges based on their CSA, and the percentage of total fibers in each range was calculated for individual mouse samples. The mean percent area for each range was determined from replicate samples.

### 2.7 Gene expression analysis by qRT‐PCR

Frozen muscles were cut into small pieces, and a 15 mg sample of each gastrocnemius, tibialis anterior, diaphragm, and heart was used for total RNA extraction using Trizol (Thermo Fisher Scientific). Muscle samples were lysed using the TissueLyser II (Qiagen). Samples were clarified by centrifugation and further processed using the RNeasy Mini Kit that included a DNase digest following the manufacturer's instructions (Qiagen). RNA was quantified by spectrophotometry (Nanodrop, Thermo Scientific). Five hundred nanograms of purified total RNA per sample was used for cDNA synthesis using Reverse Transcription cDNA Synthesis Kit (Thermo Fisher Scientific) following the manufacturer's instructions. Ten nanograms of cDNA was analyzed with gene‐specific primers for qRT‐PCR using SYBR Green PCR master mix (Applied Biosystems). qRT‐PCR was performed and gene expression analyzed on an Applied Biosystems 7500 Real‐time PCR system. *Gapdh* expression was used as an internal control. The fold change of gene expression level was analyzed using the 2^−∆∆Ct^ method.^33^

### 2.8 Metal‐ion analysis in muscles

Gastrocnemius and diaphragm muscles (15‐20 mg) were analyzed for metal‐ion concentrations at Michigan State University. Following overnight drying at 75°C, tissues were digested overnight in a 10‐fold volume of nitric acid relative to tissue dry mass. Digested samples were then diluted in a 100‐fold volume of water. Elemental analysis was performed by Inductively Coupled‐Plasma Mass Spectrometry (Agilent). Elemental concentrations were calculated utilizing a four‐point linear curve of the analyte‐internal standard response ratio with standards from Inorganic Ventures.

### 2.9 Immunoblotting

Whole frozen gastrocnemius muscles were first cut into small pieces. A representative sample of 15 mg per mouse was homogenized in 250 µL of lysis buffer (150 mmol/L NaCl, 1% NP‐40, 0.5% sodium deoxycholate, 0.1% SDS, and 50 mmol/L Tris pH 8) supplemented with 1× protease inhibitor cocktail (Roche) and 1× phosphatase inhibitor cocktail (Thermo Scientific). Muscles were homogenized for two cycles of 3 minutes using the TissueLyser II (Qiagen). Lysates were clarified by centrifugation at 15 000 × *g* for 15 minutes at 4°C, and the protein concentration was determined by BCA (Pierce Protein Assay Kit, Thermo Scientific). Proteins were resolved by SDS‐PAGE and further transferred to nitrocellulose membranes. Membranes were blocked for 1 hour at room temperature with 5% non‐fat milk in TBS‐T (25 mmol/L Tris‐HCl pH 7.4, 150 mmol/L NaCl, and 0.1% Tween 20). Membranes were incubated with the following antibodies: Phospho‐SMAD2 (Ser465/467, #3108, Cell Signaling) (1:500); SMAD2 (#5339, Cell Signaling) (1:500); followed by incubation with secondary goat anti‐rabbit and tertiary anti‐goat HRP‐conjugated antibodies (Sigma). The membranes were developed using ECL substrate for chemiluminescence (Pierce). The membranes were also incubated with skeletal actin antibody (#A2172, Sigma) (1:5000) followed by anti‐mouse‐HRP‐conjugated secondary antibody (Sigma) as an internal control. The immunoblots were visualized on a BioRad ChemiDoc Touch Imaging System.

### 2.10 Statistical analysis

All statistical analyses were performed by the unpaired, two‐tailed Student's *t* test using GraphPad Prism 8. All values were determined as the mean ± SEM *P* values < .05 were considered statistically significant.

## 3 RESULTS

### 3.1 Metastasis and cachexia development in an orthotopic breast cancer model generated from *Bard1*‐deficient tumor cells

**FIGURE 1:**
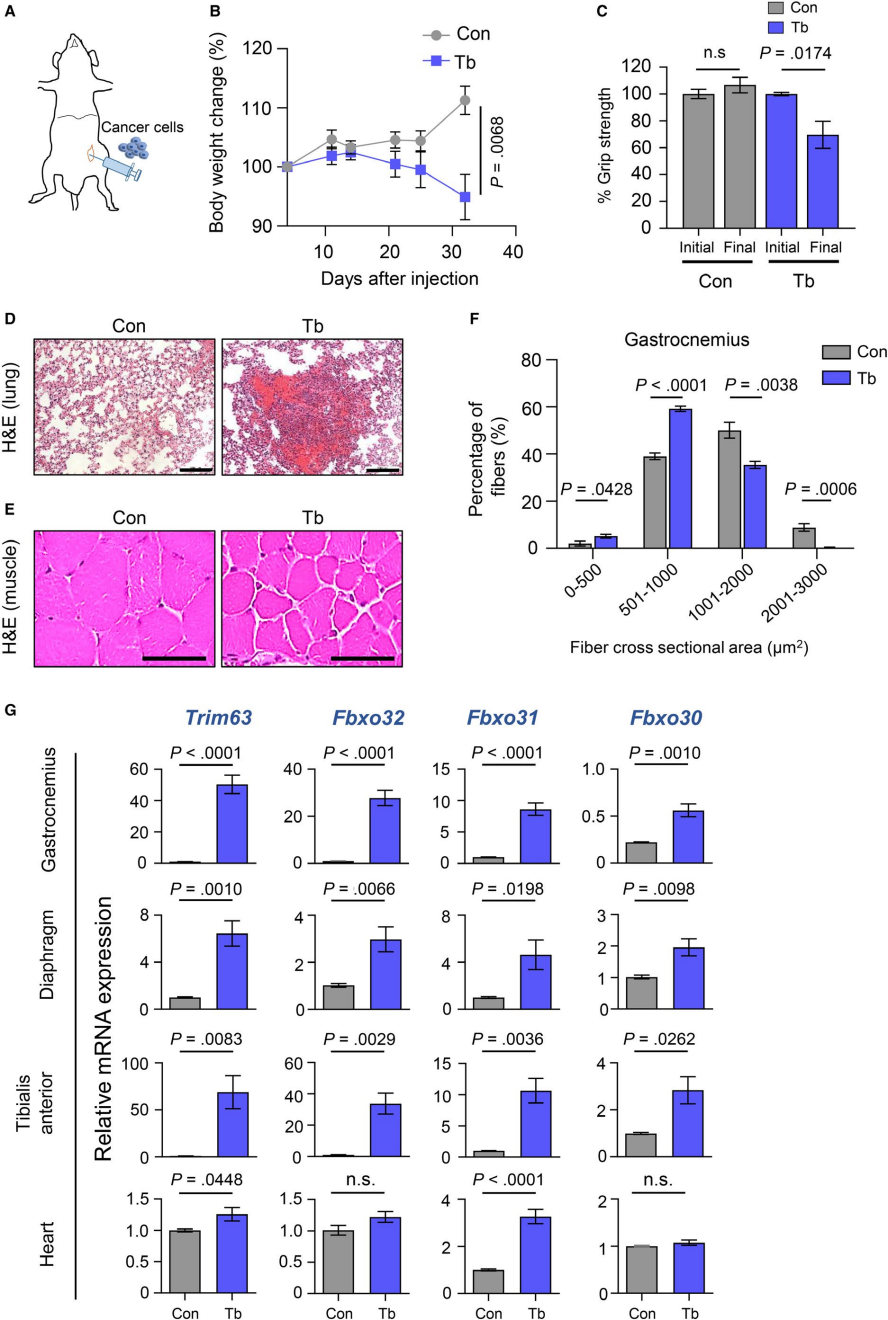
Characterization of the *Bard1*‐deficient, orthotopic model of metastasis and cachexia. A, Schematic showing orthotopic implantation of 5 × 10^5^ *Bard1*‐deficient breast cancer cells in the fourth mammary gland of syngeneic B6129SF1/J mice. B, Body‐weight analysis of nontumor‐bearing control (Con) and tumor‐bearing (Tb) mice following tumor‐cell injection. Whole body weight included tumor weights at all time points shown in the figure. Tumor growth curve shown in Figure S1. n = 5 Con mice and n = 5 Tb mice. C, Measurements of hind limb grip strength in Con and Tb mice following tumor‐cell injection (normalized to mean of initial values of each group). n = 5 Con mice and n = 5 Tb mice. D, Representative hematoxylin and eosin (H&E)‐stained images of lung tissue sections from Con and Tb mice. Scale bars represent 100 µm. E, Representative H&E‐stained images of gastrocnemius muscle cross‐sections from Con and Tb mice. Scale bars represent 50 μm. F, Quantitation of myofiber cross‐sectional areas in gastrocnemius muscles from Con and Tb mice. n = 5 Con mice and n = 5 Tb mice. G, Quantitative real‐time reverse transcription PCR (qRT‐PCR) analysis of muscle atrophy markers *Trim63*, *Fbxo32*, *Fbxo31,* and *Fbxo30* in gastrocnemius, diaphragm, tibialis anterior, and cardiac muscles. Mouse *Gapdh* gene was used as internal control. A minimum of n = 4 Con mice and n = 4 Tb mice were analyzed. All data are represented as the mean ± SEM *P* values were determined by the two‐tailed, unpaired Student's *t* test. n.s. not significant

To develop an orthotopic model of triple‐negative breast cancer, we injected 5 × 10^5^ *Bard1‐*deficient tumor cells into the fourth mammary fat pad of 8‐ to 9‐week‐old, female, syngeneic B6129SF1/J mice (Figure 1A). Tumor growth coincided with marked body weight loss (Figure 1B and Figure S1), grip‐strength reduction (Figure 1C), and lung metastasis (Figure 1D) in the tumor‐bearing mice compared to age‐matched control mice at end point. The loss of muscle strength in tumor‐bearing mice was also accompanied by a significant reduction in muscle size (muscle atrophy) as determined by morphometric analysis of myofiber CSA of the gastrocnemius muscles (Figure 1E,F). Our studies show that tumor‐bearing mice exhibited a higher percentage of fibers with smaller CSA (fiber CSA 0‐500 µm^2^: *P* = .0428 and 501‐1000 µm^2^: *P < *.0001) and a lower percentage of fibers with larger CSA (fiber CSA 1001‐2000 µm^2^: *P* = .0038 and 2001‐3000 µm^2^: *P = *.0006), compared to control mice (Figure 1F). Interestingly, genes encoding ubiquitin ligases (*Trim63, Fbxo32/MAFBx, Fbxo31,* and *Fbxo30/Musa1)* that target muscle proteins and serve as markers of muscle atrophy were found to be transcriptionally upregulated in the gastrocnemius muscles from the tumor‐bearing mice (Figure 1G). This was also observed in other skeletal muscles (diaphragm and tibialis anterior), and two of the ubiquitin ligases (*Trim63* and *Fbxo31*) were significantly upregulated (*P* = .0448 and *P* < .0001, respectively) in heart muscle (Figure 1G). In line with these findings, other genes associated with the ubiquitin proteasome and autophagy pathways were also upregulated in the cachectic gastrocnemius and diaphragm muscles (Figure S2). Thus, the *Bard1*‐deficient breast cancer metastasis model develops skeletal and cardiac muscle atrophy systemically affecting multiple muscle groups.

### 3.2 The metal‐ion transporter gene, *Zip14,* is upregulated in cachectic muscles from the *Bard1*‐deficient, orthotopic breast cancer metastasis model

**FIGURE 2:**
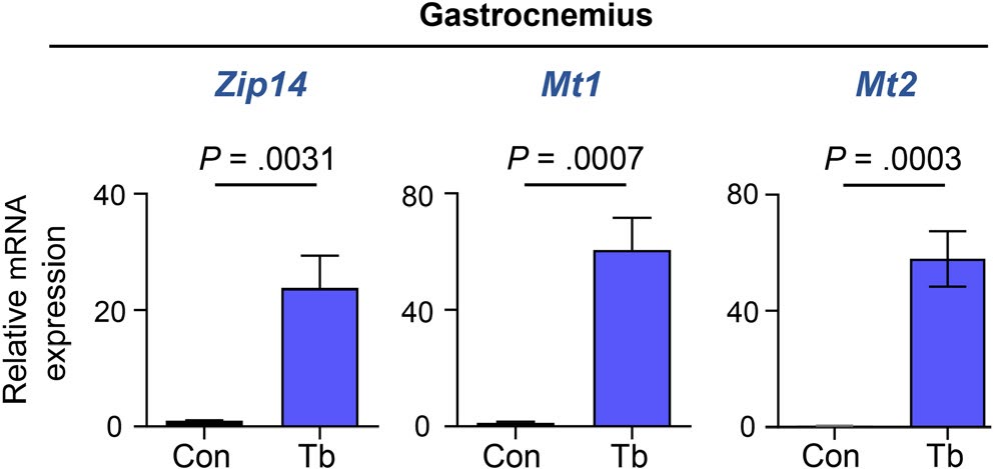
The metal‐ion transporter gene *Zip14* is upregulated in cachectic gastrocnemius muscles from the *Bard1*‐deficient, orthotopic breast cancer metastasis model. qRT‐PCR was performed to analyze the mRNA expression of *Zip14* and metallothionein 1 and 2 (*Mt1* and *Mt2*) in gastrocnemius muscles of control and tumor‐bearing mice. Mouse *Gapdh* gene was used as internal control. n = 5 Control (Con) mice and n = 5 tumor‐bearing (Tb) mice. All data are represented as the mean ± SEM *P* values were determined by the two‐tailed, unpaired Student's *t* test

**FIGURE 3:**
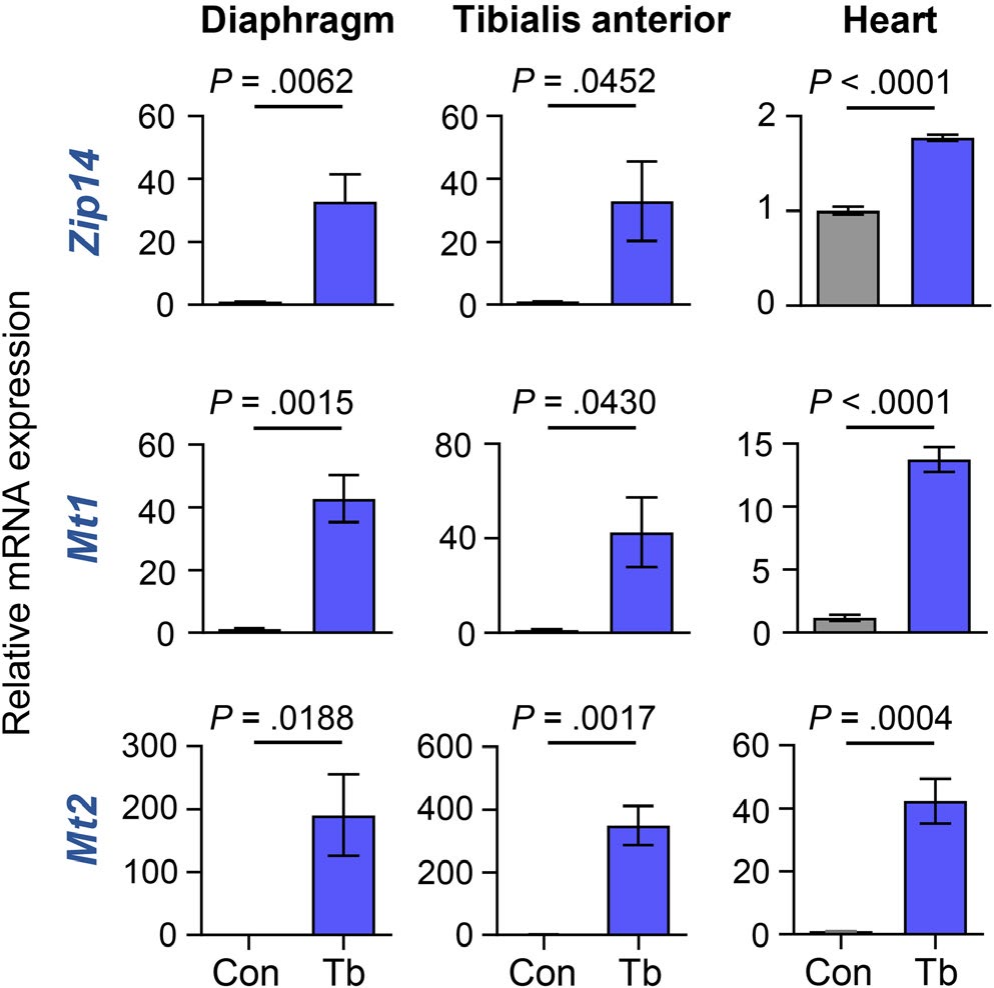
*Zip14* is upregulated in the diaphragm, tibialis anterior, and heart muscles from the *Bard1*‐deficient, orthotopic breast cancer metastasis model. qRT‐PCR was performed to analyze the mRNA expression of *Zip14, Mt1,* and *Mt2* in diaphragm, tibialis anterior, and heart muscles of control and tumor‐bearing mice. Mouse *Gapdh* gene was used as internal control. All data are represented as the mean ± SEM *P* values were determined by the two‐tailed, unpaired Student's *t* test from a minimum of four control (Con) and four tumor‐bearing (Tb) mice

Studies have revealed that the metal‐ion transporter, ZIP14, is a critical mediator of cachexia in metastatic cancer models.^25^ We therefore investigated whether cachexia development in the *Bard1*‐deficient, orthotopic metastasis model is associated with the upregulation of *Zip14* in muscle. Indeed, we found that Z*ip14* is aberrantly upregulated in the cachectic gastrocnemius muscles in these mice (Figure 2). Moreover, we observed a concomitant upregulation of the zinc‐inducible metallothionein 1 and 2 (*Mt1* and *Mt2*) genes (Figure 2), which encode cysteine‐rich, metal‐binding proteins that help maintain cellular zinc homeostasis^34^ and are upregulated in muscles undergoing atrophy.^35^, ^36^ Importantly, *Zip14, Mt1, and Mt2* expression were also significantly upregulated in diaphragm, tibialis anterior, and cardiac muscles of tumor‐bearing mice compared to control mice (Figure 3). These results are consistent with perturbed zinc homeostasis in multiple cachectic muscles, including those involved in breathing (diaphragm), circulation (cardiac muscles), and locomotion (gastrocnemius and tibialis anterior muscles), in the *Bard1*‐deficient, orthotopic metastasis model.

### 3.3 *Zip14* upregulation is associated with increased zinc levels in cachectic muscles

**FIGURE 4:**
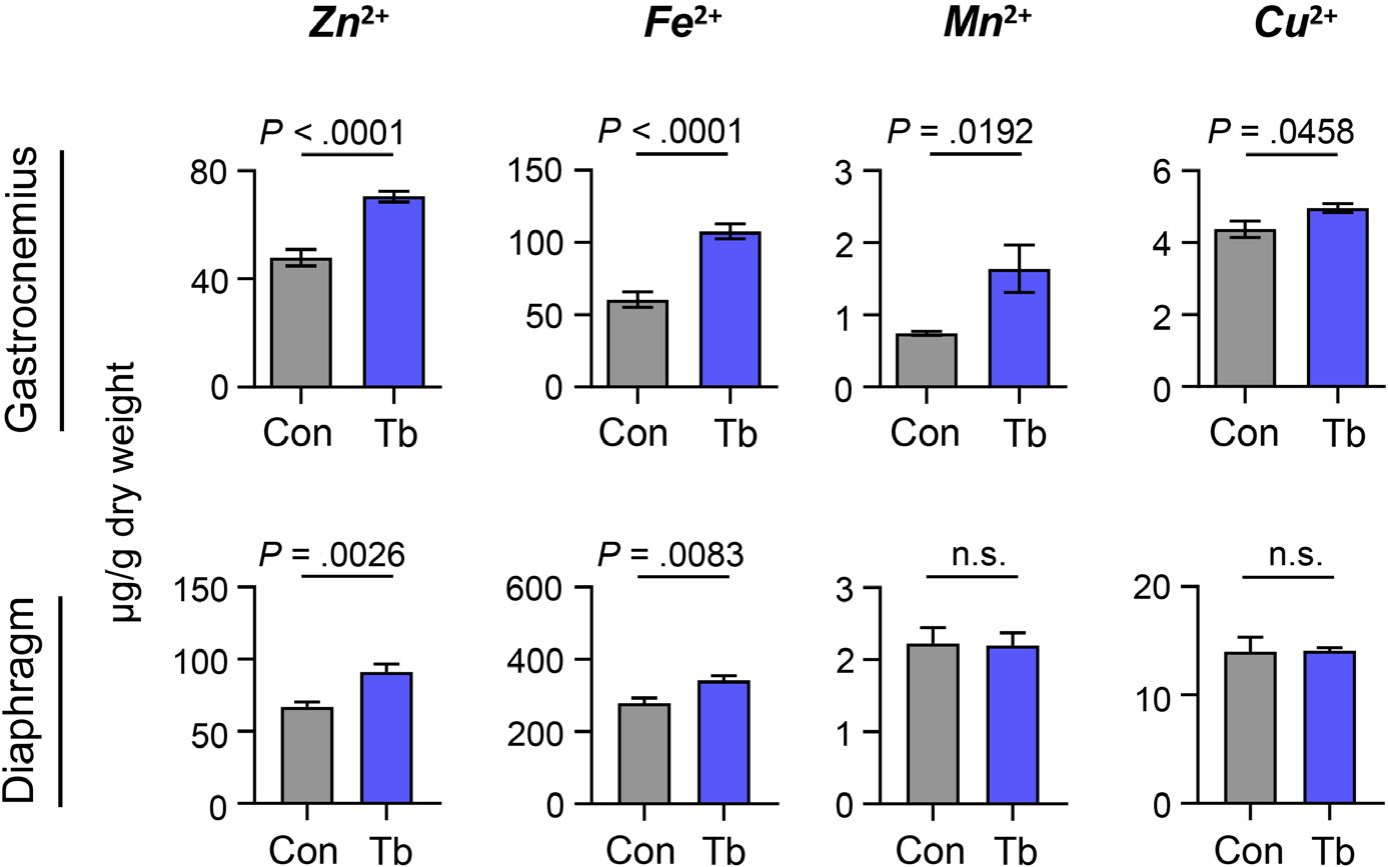
Zinc and iron levels are increased in cachectic muscles from the *Bard1*‐deficient, orthotopic breast cancer metastasis model. Metal‐ion analysis of zinc (Zn^2+^), iron (Fe^2+^), manganese (Mn^2+^), and copper (Cu^2+^) was performed on gastrocnemius and diaphragm muscles by ICP‐MS at end point and represented as µg/g of muscle dry weight. n = 7 Control (Con) mice and n = 7 tumor‐bearing (Tb) mice for gastrocnemius muscles. n = 7 Con mice and n = 6 Tb mice for diaphragm muscles. All data are represented as the mean ± SEM *P* values were determined using the two‐tailed, unpaired Student's *t* test. n.s. not significant

As a broad‐scope metal‐ion transporter, ZIP14 is important for transporting free zinc, iron, and manganese into cells.^34^, ^37^ Therefore, we tested whether *Zip14* expression in cachectic muscles of tumor‐bearing mice was associated with alterations in the levels of these metal‐ions. Gastrocnemius and diaphragm muscles were chosen as representative muscles for the analysis of metal content by inductively coupled‐plasma mass spectrometry (ICP‐MS). As shown in Figure 4, higher zinc (Zn^2+^) and iron (Fe^2+^) were detected in both tumor‐bearing gastrocnemius and diaphragm muscles compared to muscles from control mice. Manganese (Mn^2+^) levels were only increased in the gastrocnemius muscles of tumor‐bearing mice, and minor to no changes in copper (Cu^2+^) were observed between control and tumor‐bearing mice. These studies suggest that increased muscle‐cell expression of *Zip14* leads to aberrant metal‐ion levels in cachectic muscles from the *Bard1*‐deficient, orthotopic metastasis model.

### 3.4 Increased SMAD signaling coincides with *Zip14* upregulation in cachectic muscles

**FIGURE 5:**
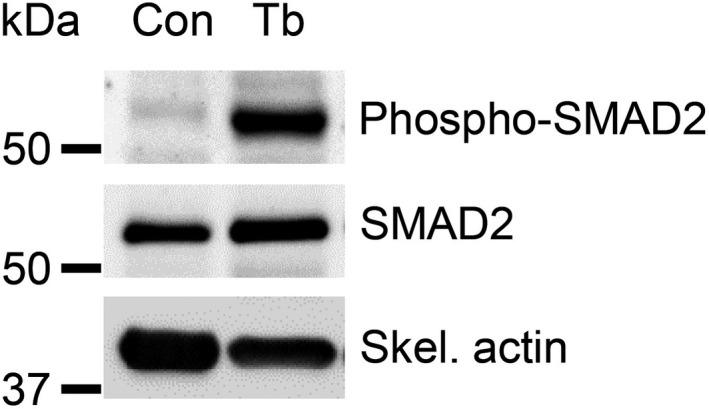
Increased phospho‐SMAD2 levels in cachectic muscles from the *Bard1*‐deficient, orthotopic breast cancer metastasis model. Immunoblot analysis was performed to detect phospho‐SMAD2, SMAD2, and skeletal actin (internal control) in gastrocnemius muscles from control (left) and tumor‐bearing mice with *Bard1*‐deficient tumors (right)

Studies have reported that the transforming growth factor‐beta (TGF‐β) signaling pathway induces *Zip14* expression in muscle cells through phosphorylation and activation of downstream SMAD effectors.^25^ To examine the possibility that the SMAD/TGF‐β signaling pathway drives increased expression of *Zip14* in the *Bard1*‐deficient model (Figure 5), we performed immunoblot analysis using antibodies against phospho‐SMAD2, SMAD2, and skeletal actin. Indeed, we observed that higher *Zip14* expression (Figure 2) coincides with greater SMAD2 phosphorylation in the cachectic gastrocnemius muscles of tumor‐bearing mice compared to control (Figure 5). This suggests that activation of the SMAD/TGF‐β signaling pathway is associated with increased *Zip14* expression in cachectic muscles from the *Bard1*‐deficient, orthotopic metastasis model.

## 4 DISCUSSION

In this study, we report and characterize a new orthotopic model of breast cancer with spontaneous metastasis. Injection of *Bard1*‐deficient tumor cells into the mammary fat pad of syngeneic mice led to spontaneous lung metastasis and gradual cachexia development, generating a useful resource for analyzing the mechanisms that underlie both metastatic progression and muscle wasting in breast cancer. However, it remains to be determined whether metastatic breast cancer patients with either *BRCA1/BARD1* mutations or triple‐negative disease with dysfunctional BRCA1/BARD1 also develop muscle mass loss and/or muscle weakness. Future clinical studies using longitudinal CT‐imaging analysis of adipose tissue and muscle mass in *BRCA1/BARD1*‐mutant breast cancer patients are awaited to provide this key information.

The *Bard1*‐deficient, orthotopic model of breast cancer metastasis will be a useful preclinical model for translational studies. For instance, the dysfunction of BRCA1/2 and/or BARD1 sensitizes cancer cells to chromosomal instability and subsequent apoptosis induced by inhibition of poly (ADP‐ribose) polymerase (PARP) enzymatic activity.^38^ PARP inhibitors are approved for the treatment of breast cancer patients with inherited cancer‐associated BRCA1/2 mutations, and ongoing clinical trials are evaluating the efficacy of PARP inhibitors for the treatment of sporadic, triple‐negative breast cancers that share similarities, or “BRCA‐ness,” with inherited BRCA1/2‐mutant breast cancers.^39^, ^40^ Therefore, our newly characterized *Bard1*‐deficient, orthotopic metastasis model can be used to accelerate the evaluation of PARP inhibitor efficacy in metastatic breast cancers harboring sensitizing BRCA1/2 pathway mutations. Interestingly, PARP activity may also promote cachexia. PARP activation occurs in the muscles of mice that develop lung cancer,^41^ and deletion of either PARP1 or PARP2 reduces body weight loss, decreases the expression of genes involved in muscle proteolysis, and promotes the expression of anabolic markers in lung cancer‐induced cachexia.^41^ Although the effect of PARP inhibition on muscles remains elusive in the context of breast cancer, the *Bard1*‐deficient, orthotopic model will provide new opportunities to address this important question.

In this study we show that systemic upregulation of *Zip14* in muscle is associated with severe cachexia in the *Bard1*‐deficient, orthotopic model. Interestingly, both zinc and iron levels were increased in cachectic muscles in this model, an observation that is distinct from colon and lung cancer models in which only zinc was elevated in cachectic muscles with high *Zip14* expression. It is unclear whether ZIP14 exclusively transports both zinc and iron into muscle cells or if other metal‐ion transporters also contribute to this process in the context of cancer‐induced cachexia. Nonetheless, our results indicate that ZIP14 inhibitors and chelation strategies for zinc and iron^42^, ^43^ should be tested in future studies to restore metal‐ion homeostasis in cachectic muscles. Based on reported studies^25^ and our current findings, we expect that blocking ZIP14‐mediated myofiber zinc accumulation or incorporating metal‐ion chelation strategies will reduce cachexia in the *Bard1*‐deficient, orthotopic model. Future preclinical studies will be necessary to test whether combining these anticachexia therapies with antineoplastic agents can restore muscle strength and prolong survival in cachectic cancer patients.

It will be important to determine which factors mediate the upregulation of *Zip14* expression in muscle cells in the *Bard1*‐deficient, orthotopic model in future studies. It has previously been reported that elevated TGF‐β signaling induces *Zip14* expression in‐vitro and that the inhibition of TGF‐β‐induced SMAD phosphorylation with a TGF‐β RI kinase inhibitor reduces *Zip14* expression.^25^ Furthermore, it is important to note that the neutralization of circulating TGF‐β with a pan‐TGF‐β antibody in cachectic 4T1 breast and C26m2 colon cancer mouse models reduces SMAD2 phosphorylation and *Zip14* expression in muscles.^25^ Since we found that SMAD2 phosphorylation is significantly higher in cachectic muscles from the *Bard1*‐deficient, orthotopic model compared to control muscles, it is plausible that the TGF‐β pathway regulates *Zip14* expression during breast cancer metastasis and that the new class of TGF‐β pathway inhibitors that is currently being tested in clinical trials^44^ could be useful for the treatment of breast cancer‐associated cachexia. Alternatively, increased *Zip14* expression in cachectic muscles could also lead to SMAD2 activation through unknown mechanisms. Our present studies do not distinguish between these possibilities and show an association of the SMAD2 activation and *Zip14* expression in cachectic muscles in the Bard1‐deficient, orthotopic model. It is important to note that a number of inflammatory cytokines, such as TNF‐α, have been shown to induce *Zip14* expression in muscle cells.^25^ Knowledge of the specific factors that regulate *Zip14* induction in muscle cells in the *Bard1*‐deficient, orthotopic model could therefore inform new therapeutic opportunities to prevent or reverse cachexia in triple‐negative breast cancer models with disrupted *Brca1/Bard1* function.

## CONFLICTS OF INTEREST

The authors declare no conflict of interest.

## AUTHOR CONTRIBUTIONS

ARS, TJZ, WM, CC, RH, HS, and VC performed the experiments. ARS and WM generated the figures. MS, KT, and H.H performed histopathological analysis. RB provided the Bard1‐deficient breast cancer cell line. AKB and SA designed the study and wrote the manuscript. SA supervised the study. All the authors read and reviewed the manuscript and approved the study.

## Supporting information

Fig S1

Fig S2

## Data Availability

All data from this study will be made available upon request from the corresponding author. Information on all resources (eg, assay protocols, cell lines) will be shared via publication in scholarly journals, NCBI repository, and at scientific meetings. Any proprietary materials that arise from this work will be shared to academic researchers via Materials Transfer Agreements through the Office of Technology Commercialization at Columbia University.
