# Supplementary figures and images for: Aberrant Zip14 expression in muscle is associated with cachexia in a Bard1‐deficient mouse model of breast cancer metastasis
Source: Cancer Med. 2020 Jul 30;9(18):6766–75. doi: 10.1002/cam4.3242 (PMC7520359; doi:10.1002/cam4.3242)

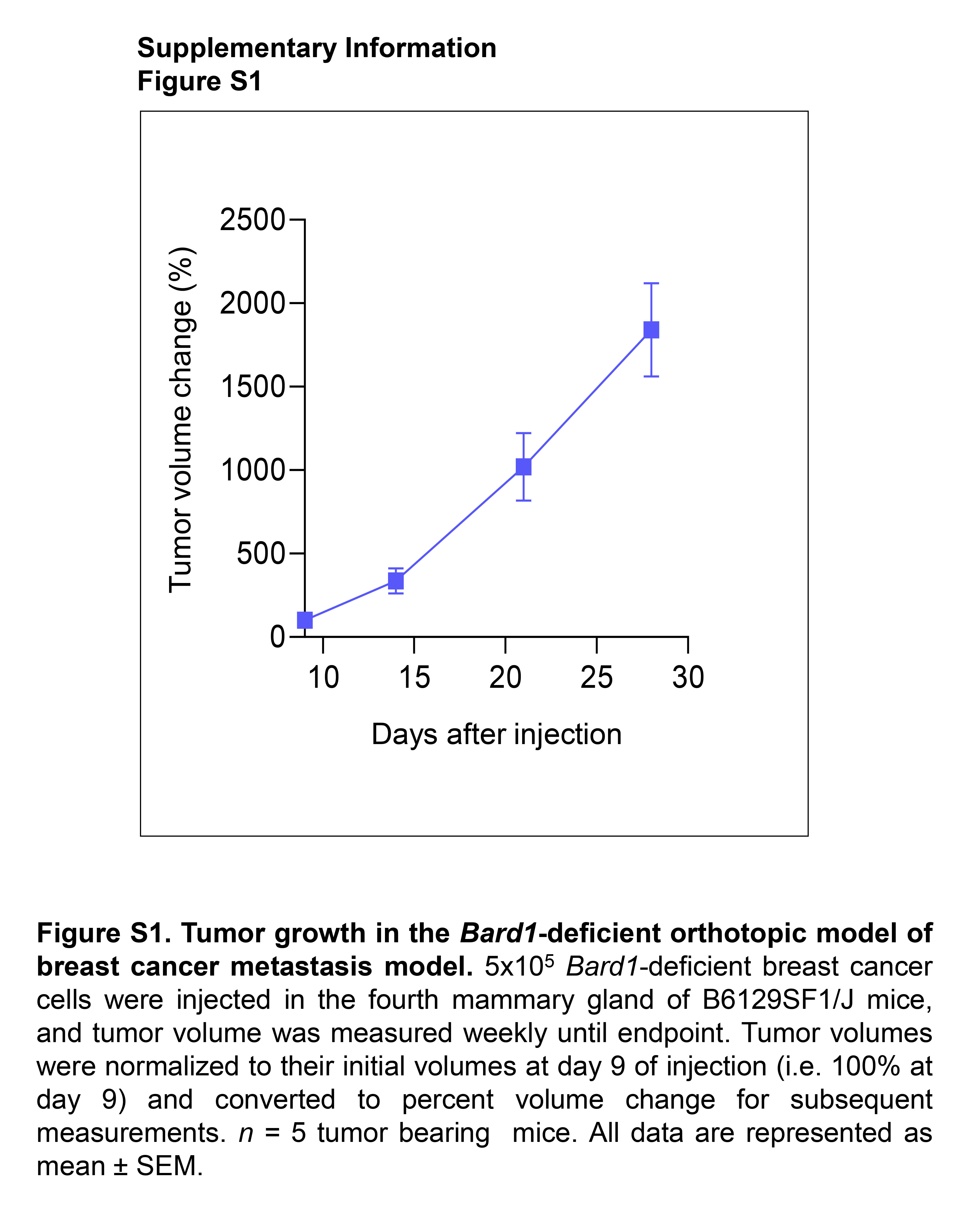

Supplement: Supplementary file 1 — Fig S1 [file CAM4-9-6766-s001.jpg]

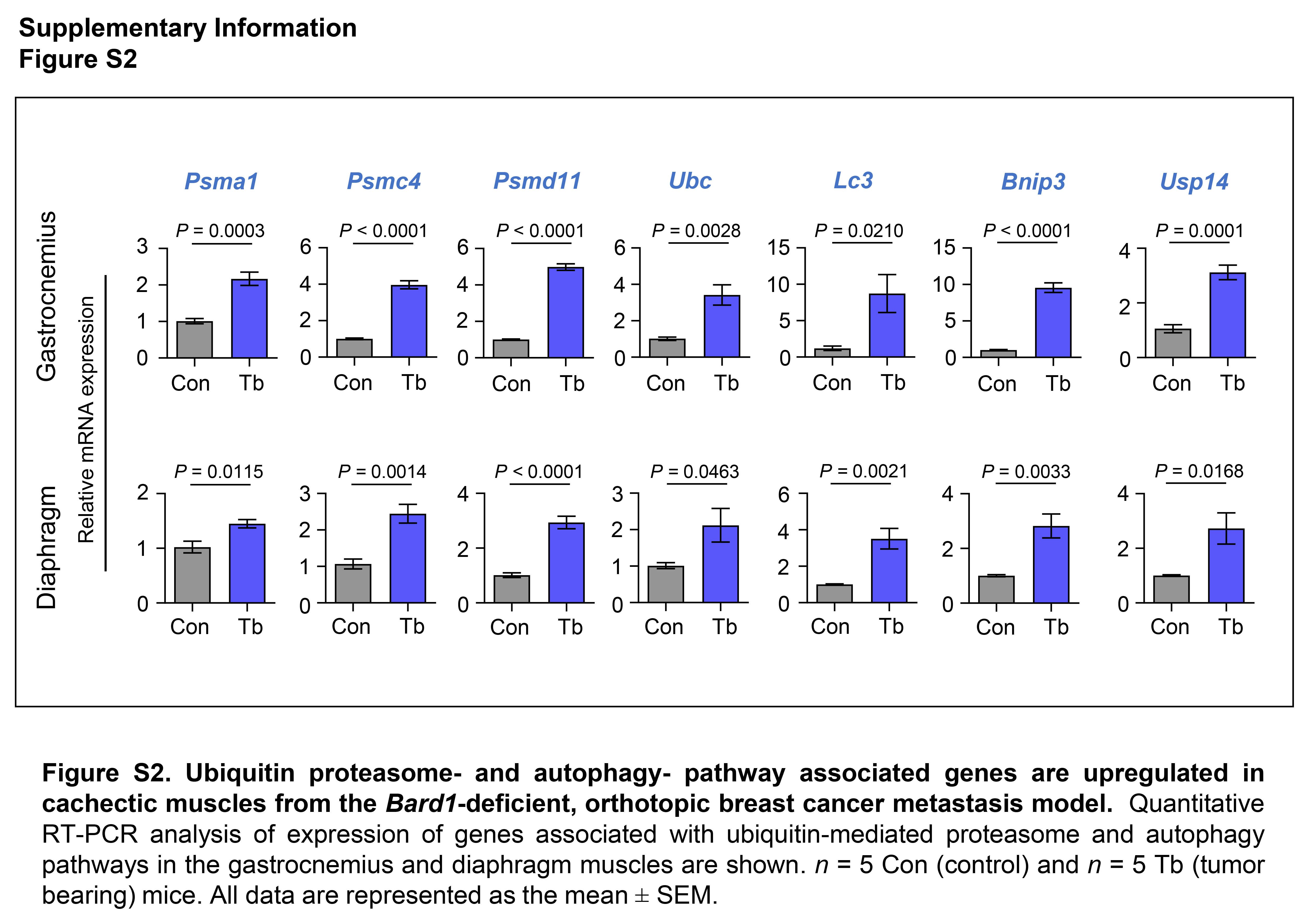

Supplement: Supplementary file 2 — Fig S2 [file CAM4-9-6766-s002.jpg]
